# CS-GA-XGBoost-Based Model for a Radio-Frequency Power Amplifier under Different Temperatures

**DOI:** 10.3390/mi14091673

**Published:** 2023-08-27

**Authors:** Jiayi Wang, Shaohua Zhou

**Affiliations:** 1 School of Micro-Nano Electronics, Zhejiang University, Hangzhou 310058, China; 22241067@zju.edu.cn; 2 ZJU-Hangzhou Global Scientific and Technological Innovation Center, Zhejiang University, Hangzhou 310058, China; 3 Qingdao Institute for Marine Technology of Tianjin University, Qingdao 266200, China; 4 Research Center for Intelligent Chips and Devices, Zhejiang Lab, Hangzhou 311121, China

**Keywords:** XGBoost, cuckoo search, genetic algorithm, modeling, power amplifier

## Abstract

Machine learning methods, such as support vector regression (SVR) and gradient boosting, have been introduced into the modeling of power amplifiers and achieved good results. Among various machine learning algorithms, XGBoost has been proven to obtain high-precision models faster with specific parameters. Hyperparameters have a significant impact on the model performance. A traditional grid search for hyperparameters is time-consuming and labor-intensive and may not find the optimal parameters. To solve the problem of parameter searching, improve modeling accuracy, and accelerate modeling speed, this paper proposes a PA modeling method based on CS-GA-XGBoost. The cuckoo search (CS)-genetic algorithm (GA) integrates GA’s crossover operator into CS, making full use of the strong global search ability of CS and the fast rate of convergence of GA so that the improved CS-GA can expand the size of the bird nest population and reduce the scope of the search, with a better optimization ability and faster rate of convergence. This paper validates the effectiveness of the proposed modeling method by using measured input and output data of 2.5-GHz-GaN class-E PA under different temperatures (−40 °C, 25 °C, and 125 °C) as examples. The experimental results show that compared to XGBoost, GA-XGBoost, and CS-XGBoost, the proposed CS-GA-XGBoost can improve the modeling accuracy by one order of magnitude or more and shorten the modeling time by one order of magnitude or more. In addition, compared with classic machine learning algorithms, including gradient boosting, random forest, and SVR, the proposed CS-GA-XGBoost can improve modeling accuracy by three orders of magnitude or more and shorten modeling time by two orders of magnitude, demonstrating the superiority of the algorithm in terms of modeling accuracy and speed. The CS-GA-XGBoost modeling method is expected to be introduced into the modeling of other devices/circuits in the radio-frequency/microwave field and achieve good results.

## 1. Introduction

As an essential part of wireless communication systems, the performance of radio-frequency (RF) power amplifiers (PA) play an important role in wireless communication systems [1]. Modeling of RF PA is crucial for wireless communication [2,3,4].

Various modeling methods have been widely used in the past decades to model RF PA [5]. As is well known, neural networks (NNs) are commonly used in modeling due to their learning ability based on appropriate errors and good nonlinear function fitting ability [6]. The modeling method based on a real-value delay neural network (RVTDNN) uses backpropagation as the network training algorithm, effectively describing the nonlinear characteristics of PA [7]. Using Chebyshev polynomials in the complex field as the input of the neural network, the functional link neural network (FLNN) is also used for PA modeling. Compared with the real-valued neural network (RVNN), the FLNN has a faster rate of convergence speed and lower computational complexity [8]. By converting one-dimensional signal data into two-dimensional data, a convolutional neural network (CNN) can significantly reduce the number of coefficients, reduce the training complexity, and improve the speed of PA modeling [9]. Although the neural network has shown significant accuracy in prediction, it lacks interpretability. In addition, the neural network performs poorly in processing relatively small datasets, limiting their application in PA modeling, as most PAs cannot provide sufficient massive data for model training [10].

Machine learning algorithms also have good performance in predicting classification and regression problems. Moreover, the requirement for the size of the dataset is not high, and better predictions can be obtained in a relatively short period [10]. Random forest has been introduced into PA modeling [11,12]. The forest is composed of many random trees, having high randomness and strong generalization ability, reducing the occurrence of model overfitting and thus improving the model’s accuracy [11,12]. However, high randomness comes at the cost of increasing the number of trees in the forest, increasing the computational burden and modeling time [13]. Results of PA modeling by machine learning algorithms, such as gradient boosting, K-nearest neighbor (KNN), random forest, and decision trees, are compared, and the conclusion has been drawn that gradient boosting has the best modeling accuracy and fastest speed. However, due to the slight variance of the base model used in gradient boosting, the variance of the overall model increases with serial iterative training, resulting in a weak ability of the model to prevent overfitting [13]. Chen et al. [14] proposed XGBoost in 2016. XGBoost effectively limits the complexity and variance of the model and prevents overfitting by introducing a regularization term into the loss function of gradient boosting [15]. To ensure optimal algorithm performance, optimization techniques must be used to determine the critical parameters in XGBoost: learning rat; max_depth; and n_estimators [16]. The traditional parameter optimization technique based on a grid search is time-consuming and labor-intensive. It is more common to use swarm intelligence optimization techniques [16], such as a genetic algorithm (GA) and cuckoo search (CS). Jiang et al. first introduced GA into XGBoost for training parameters and demonstrated its high accuracy in pedestrian detection [17]. In addition, Yu et al. introduced the GA-XGBoost method to disease risk and cost prediction and showed that GA-XGBoost could improve prediction accuracy [18]. Although GA has a fast convergence rate that can help find the optimal hyperparameters faster and more accurately, it is prone to mature [19]. Zhang et al. introduced CS into XGBoost in 2022 for an insulator pollution degree detection problem and found it could improve classification accuracy [20]. Though CS has few parameters and its convergence rate is not sensitive to parameter changes, it does not easily fall into local optimization. However, its convergence is still slow and lacks vitality [21]. It can be seen that a single swarm intelligence optimization search technique has its shortcomings.

For this reason, this paper proposes a modeling method of CS-GA-XGBoost, integrating GA’s crossover operator into CS, making full use of the advantages of CS’s strong global search ability and GA’s fast rate of convergence [19,21], and applying it to XGBoost’s optimal parameter search, to achieve a more rapid rate of convergence and optimization ability. This paper validates the effectiveness of the proposed CS-GA-XGBoost using measured data of the 2.5-GHz-GaN class-E PA and 2.2–6.5-GHz CMOS PA under three different temperatures (−40 °C, 25 °C, and 125 °C). The experimental results show that compared to XGBoost, GA-XGBoost, and CS-XGBoost, the modeling accuracy of CS-GA-XGBoost improved by one order of magnitude or more, and the modeling time shortened by one order of magnitude or more. In addition, compared with classic machine learning algorithms, including gradient boosting, random forest, and support vector regression (SVR), the proposed CS-GA-XGBoost can improve modeling accuracy by three orders of magnitude or more and shorten modeling time by two orders of magnitude, proving the superiority of the proposed CS-GA-XGBoost in terms of modeling accuracy and speed.

The rest of the paper is organized as follows. Section 2 provides a detailed introduction to the principle, advantages, and modeling procedure of the CS-GA-XGBoost proposed in this paper. Section 3 provides a detailed analysis of the modeling results using CS-GA-XGBoost based on the measured data of the 2.5-GHz-GaN class-E PA and 2.2–6.5 GHz CMOS PA under three different temperatures (−40 °C, 25 °C, and 125 °C). Section 4 summarizes the overall content of this paper.

## 2. CS-GA-XGBoost

### 2.1. XGBoost

**Figure 1:**
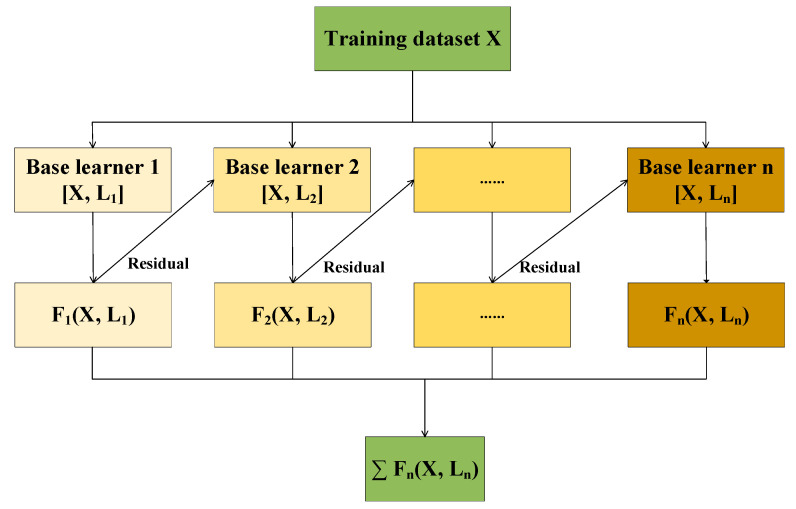
XGBoost model.

Chen et al. [22] proposed XGBoost in 2016. It is an ensemble learning algorithm based on decision trees and uses a gradient-boosting framework. The XGBoost model is shown in Figure 1. It is the same as gradient boosting. They are based on the idea of serial iterations, using the value of the negative gradient of the loss function in the current model as the approximate in the boosting tree algorithm of the regression problem and using this value as the goal to train a primary learner for collection, to update the model [23]. The difference is that the loss function in XGBoost contains a regularization term [22] to prevent overfitting, which can effectively control the complexity of the model, prevent overfitting, and improve the model’s prediction accuracy.

Given a training set $S={\left\{\left(x_{i},y_{i}\right)\right\}}_{i=1}^{N}$, gradient boosting (XGBoost) aims to minimize the loss function $L\left(y,F\left(x\right)\right)$ to find the optimal approximate solution $\hat{F}\left(x\right)$ of the function $F^{*}\left(x\right)$. Assuming the initial values $F^{*}\left(x\right)=f_{0}\left(x\right)$, and regarding $F^{*}\left(x\right)$ as a whole, the optimal function after *T* iterations is as follows [13]:

(1) $$F^{*}\left(x\right)=\sum_{t=0}^{T}f_{t}\left(x\right),$$

where $f_{t}\left(x\right)$ is as follows [13]:

(2) $$f_{t}\left(x\right)=f_{t-1}\left(x\right)+\Delta f_{t}\left(x\right)=f_{t-1}\left(x\right)-\alpha_{t}*\gamma ,$$

where $\alpha_{t}$ represents the weight of the function $f_{t}\left(x\right)$, and γ represents the parameters calculated from the negative gradient of the loss function in the current mode [13]:

(3) $$\gamma =-\left[\frac{\partial L\left(y,F\left(x\right)\right)}{\partial F\left(x\right)}\right],$$

where $L\left(y,F\left(x\right)\right)$ is the loss function. In gradient boosting, $L\left(y,F\left(x\right)\right)$ is the squared difference loss function. In XGBoost, an additional regularization term is added to prevent overfitting [13]:

(4) $$L_{XGBoost}\left(y,F\left(x\right)\right)={\left(y-F\left(x\right)\right)}^{2}+\sum_{t=1}^{T}\varphi \left(f_{t}\left(x\right)\right),$$

where $\varphi \left(f_{t}\left(x\right)\right)$ represents the additional regularization term.

Hyperparameters largely influence the performance of XGBoost. There are more than ten hyperparameters in XGBoost, but their impact on the accuracy of XGBoost varies. Three hyperparameters have the most significant impact: (1) n_estimators represent the number of trees established in the model. Typically, the larger the n_estimators are, the more accurate the model is, but it is also prone to overfitting. (2) learning_rate represents the step size when iterating the decision trees. Generally, the more significant the learning rate, the faster the iteration speed is, but it may not converge to the best. The lower the learning rate is, the more likely it is to find a more accurate optimal value, but the iteration speed slows down. (3) max_depth represents the maximum depth of the tree. Usually, the larger max_depth is, the more complex the trees are, and the better the model accuracy is, but it is prone to overfitting [15,16].

Generally, a grid search is chosen to determine these three parameters. A grid search is a technique that adopts the idea of class enumeration, which finds the optimal parameters by comparing all results within a specified parameter range. However, undoubtedly, this will significantly increase search time, and accuracy is also limited by parameters [19]. A more popular parameter search method is the meta-heuristic algorithm, introduced by Glover [24] in the tabu search (TS) algorithm in 1989. It is a product of a combination of random and local search algorithms based on computational intelligence mechanisms to solve complex problems with optimal or satisfactory solutions. Meta-heuristic optimization algorithms can be divided into three categories: evolutionary algorithms, physics-based algorithms, and swam intelligence algorithms [25].

### 2.2. Genetic Algorithm (GA)

**Figure 2:**
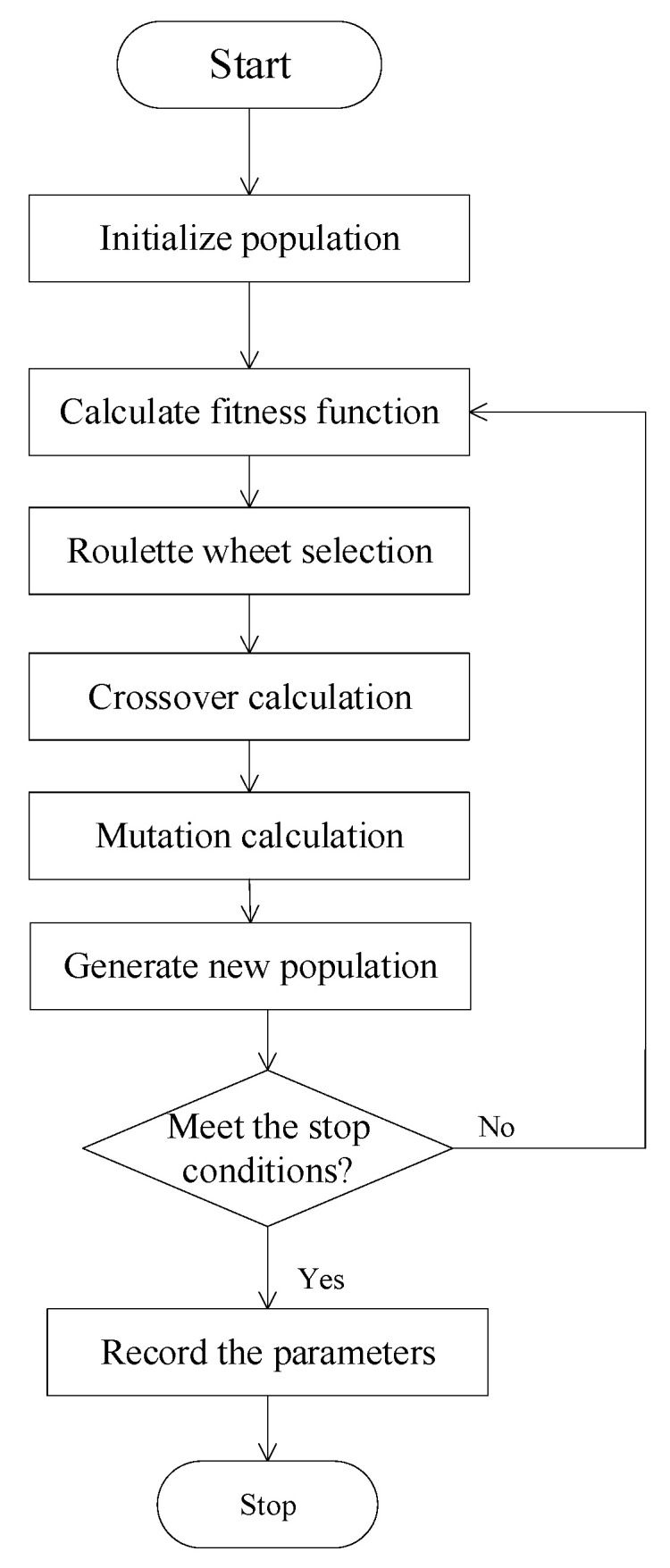
Genetic algorithm flow chart.

The concept of biological evolution in nature inspires the evolutionary algorithm. The typical evolutionary algorithm is the genetic algorithm (GA), which John Holland of the United States proposed in the 1970s [26]. Figure 2 is the flow chart of GA. The search principle of GA is based on the mechanism of natural selection and natural genetics; that is, survival of the fittest and chromosome exchange mechanism simulating biological evolution. The essential GA comprises three operators: selection; crossover; and mutation. Improved genes can be preserved through the evolution of several generations of populations. The algorithm defines a fitness function to evaluate whether genes are good enough [26]. The GA is unaffected by problem properties and optimization criteria and only utilizes objective functions for global adaptive search under guidance probability. It can handle complex problems that traditional optimization methods find challenging to solve. However, if selection methods, crossover, and mutation are inappropriate, the GA will exhibit some issues, such as being prone to premature maturation and falling into local optimization [27].

### 2.3. Cuckoo Search (CS)

**Figure 3:**
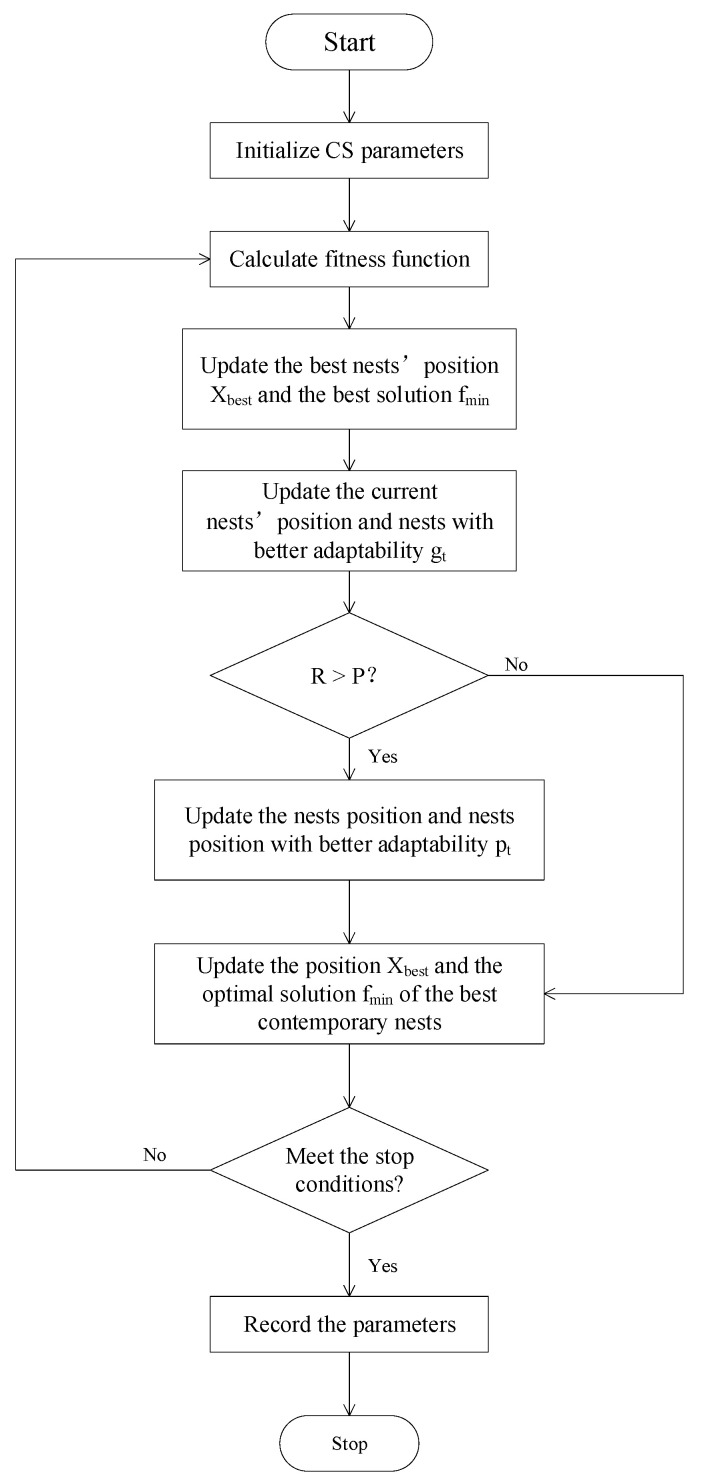
Cuckoo search flow chart.

In 1989, Gerardo Beni and Jing Wang first proposed the concept of swarm intelligence (SI) in the context of cellular robots [28]. It utilizes information exchange and cooperation between groups to achieve optimization through simple and limited personal interaction. The core idea is to simulate the behavior of insects, cattle, birds, fish, and other groups cooperatively searching for food. Each group member constantly changes the direction of their search by learning from their own experience and the experiences of other members. Cuckoo search (CS) was proposed by Yang and Deb [29] in 2009. Figure 3 is the flow chart of CS. CS is a swarm intelligence search technology that combines cuckoo nests and levy parasitism. The eggs produced by the cuckoo in the selected nest can be regarded as a solution to the problems to be solved. The eggs produced by the cuckoo in the following more optimal nest are the optimal solutions to the problem to be solved. Cuckoos searching for nests and laying eggs are seeking the optimal solution to the problem. The eggs produced by the optimal nest selected by more cuckoos will form optimal solutions [28]. Cuckoo has few search parameters, and the convergence rate is not sensitive to parameter changes, therefore it does not easily fall into local optimization and has a strong global search ability. However, its searchability is at the cost of a slow convergence rate, lacking vitality [21].

### 2.4. CS-GA

**Figure 4:**
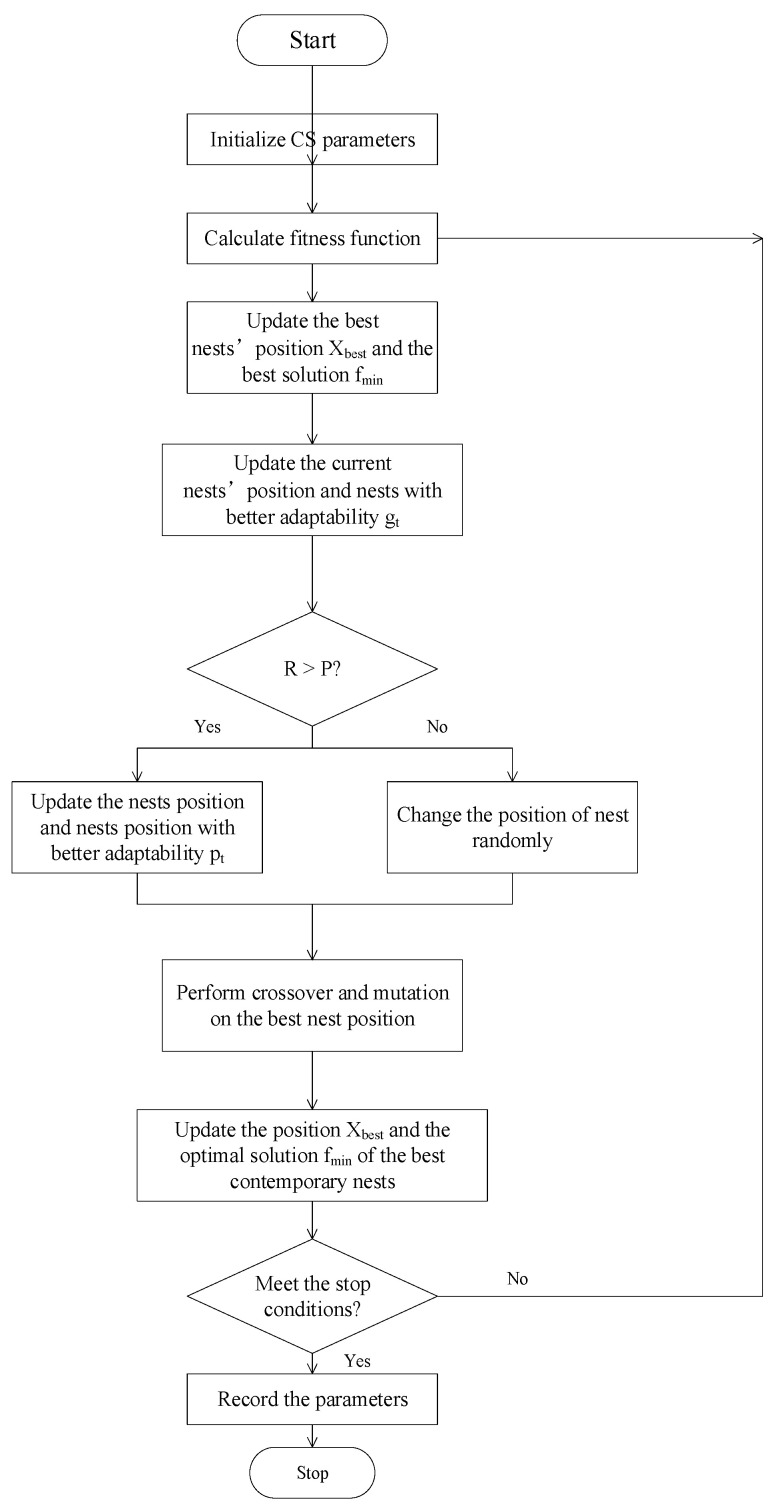
CS-GA flow chart.

Considering the advantages and disadvantages of CS and GA, this paper first proposes an optimization algorithm for CS-GA. By integrating GA’s crossover operator into CS, taking full advantage of CS’s strong global search ability and GA’s fast rate of convergence, the improved CS-GA can expand the size of the bird nest population and reduce the search range of the bird nest population, enabling the model to have a faster rate of convergence and optimization ability. The flow chart of the proposed CS-GA is shown in Figure 4. The following provide a specific description:

(1) Initialize the algorithm. Initialize parameters such as nest size N, dimension D, discovery probability P, nest boundary value lb, and ub.
(2) Calculate the fitness value of the bird’s nest.
(3) Update the optimal nest position X_best_ and the optimal solution f_min_.
(4) Update the current birds’ nest location and compare it with the previous generation to update their nest g_t_ with better adaptability.
(5) If R > P, update the birds’ nest position and compare with the birds’ nest position g_t_, and update the birds’ nest position p_t_ with better adaptability. If R < P, randomly change the position of the bird’s nest.
(6) Perform GA’s crossover and mutation on the best set of bird nest positions.
(7) Update the current optimal nest position X_best_ and the optimal solution f_min_.
(8) Determine the stop conditions. If it meets, record the parameters and end. Otherwise, return to step (2) for a new round of training.

The construction of CS is based on three ideal assumptions: (1) each cuckoo will only lay one egg at a time, and the nest will be randomly selected for laying eggs; (2) only produce the best eggs in the best nest; (3) the probability of foreign bird eggs being discovered is $P\in \left[0,1\right]$, and after being discovered by the host, they will directly abandon the nest or bird eggs. Based on these three rules, the formula for updating the location and path of cuckoos searching for their nests is [21]:

(5) $$x_{i}^{\left(t+1\right)}=x_{i}^{t}+\alpha ⊕L\left(\lambda \right),$$

where $x_{i}^{t}$ represents the position of *i*-th nest at iteration *t*. α(>0) is the step size, ⊕ is the product, $L\left(\lambda \right)$ follows the Levy distribution, essentially providing a random step size:

(6) $$Levy\left(\lambda \right)\sim \mu =t^{-\lambda }(1<\lambda \leq 3)$$

For *x*, a roulette wheel strategy is used to select individuals for cross-operation. The intersection of two individuals *x*_1_ and *x*_2_ [26]:

(7) $$x_{1}^{new}=\omega \cdot x_{1}+\left(1-\omega \right)\cdot x_{1}$$

(8) $$x_{2}^{new}=\omega \cdot x_{2}+\left(1-\omega \right)\cdot x_{2}$$

where *x*_1_ and *x*_2_ are parent individuals, while $x_{1}^{new}$ and $x_{2}^{new}$ are sub entities. ω is a random number with a weight of 0 to 1. Mutation particles are randomly selected, and the mutation operational definition is defined as follows.

(9) $$x^{new}=x+\omega \cdot \left(x_{max}-x_{min}\right)$$

where the generated $x^{new}$ is added to the birds’ nest population.

The improved CS-GA first uses CS to find the optimal location for contemporary bird nests and then propagates the elite bird nests through cross-mutation operations. On the one hand, this has expanded the size of the birds’ nest group. On the other hand, it has narrowed the search scope of the birds’ nest group. Compared with CS, CS-GA can find the optimal solution with fewer iteration rounds, so it has a faster convergence rate and optimization ability.

### 2.5. CS-GA-XGBoost

**Figure 5:**
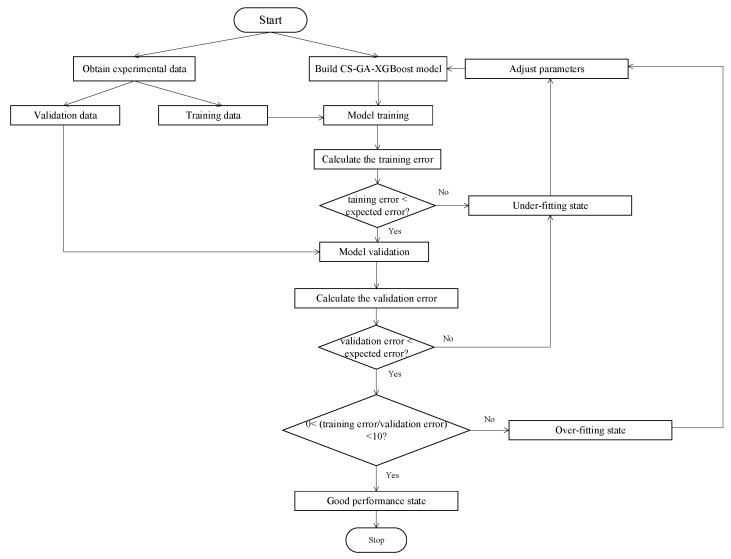
The proposed CS-GA-XGBoost modeling procedure.

Figure 5 shows the modeling procedure based on CS-GA-XGBoost. Specifically, it can be described by the following steps:

(1) Data acquisition. By measuring a 2.5-GHz-GaN class-E PA, obtain the input and output data required for modeling.
(2) Data division. Divide the obtained experimental data equally into two parts: training and validation data.
(3) Build a CS-GA-XGBoost model.
(4) Model training and calculating training errors. To evaluate the performance of different modeling techniques, mean squared error (MSE) is selected as the model accuracy evaluation standard [30]:
  (10) $$\mathrm{MSE}=\frac{1}{N}\sum_{i=1}^{N}{\left(y_{i}-{\hat{y}}_{i}\right)}^{2}$$
  where $y_{i}$ represents the actual value and ${\hat{y}}_{i}$ represents the predicted value. The smaller the MSE is, the more accurate the model is.

Suppose the training error is greater than the expected error. In that case, it indicates that the model is underfitting [31], and it is necessary to adjust the parameters and return to step (3) to rebuild the model. If the training error is less than expected, the model has completed training and entered step (5).

(5) Model validation and calculating the validation errors. Suppose the validation error is greater than the expected error. In that case, the model is underfitting [31], and the parameters must be adjusted before returning to step (3) for remodeling. Suppose the validation error is less than expected, while the difference between the training and validation errors is more significant than one order of magnitude. In that case, it indicates that the model is overfitting [31], and it is also necessary to adjust the parameters and return to step (3) to remodel. Suppose the validation error is less than the expected error, and the difference between the training and validation errors is less than one order of magnitude. In that case, the model performance is good [31], and the modeling is completed.

## 3. Results and Discussion

### 3.1. Experimental Setup

**Figure 6:**
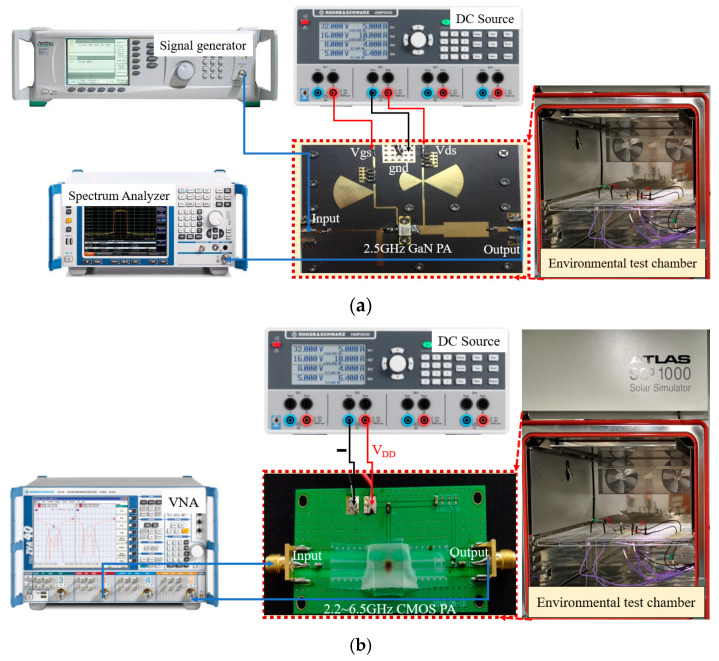
Measurement environment and connection diagram: (**a**) output power; (**b**) S_21_.

A schematic diagram of the measurement environment and connections for measuring a 2.5-GHz-GaN class-E PA at different ambient temperatures is given in Figure 6a. The 2.5-GHz-GaN class-E PA uses the CREE GaN HEMTs with a model number CGH40010F, and the selected plate was Rogers 5880 with a dielectric constant of 2.2 and a thickness of 31 mils. The whole measurement process was carried out in an environmental test chamber. A signal generator, a DC power supply, and a spectrum analyzer were used in the measurement process, whose primary roles were to provide an input signal to the PA to be tested, to provide an operating bias, and to obtain an output signal, respectively.

We measured the PA’s input and output power at three different temperatures (−40 °C, 25 °C, and 125 °C). The temperature characterization of the RF amplifier was performed in an environmental chamber (SC^3^ 1000 MHG from Vötsch Industrietechnik (Gie&szlig, Germany)) with a measured temperature range of −40 °C to +125 °C. A total of 28 data points was measured in each turn. Half of the data points in the experiment were assigned as the training data, and the rest were set as the validation data.

We also measured the S_21_ of the PA in 2.2–6.5 GHz under three different temperatures (−40 °C, 25 °C, and 125 °C). The experimental setup is shown in Figure 6b. As shown in Figure 6a, the S_21_ of the 2.2–6.5 GHz CMOS PA was also carried out in an ambient temperature chamber (SC^3^ 1000 MHG), where a DC power supply provided bias voltages and currents to the PA and a vector network analyzer (VNA) was used to measure the S_21_ of the PA. A total of 800 data points were measured in each turn. Half of the data points in the experiment were assigned as the training data, and the rest were set as the validation data.

### 3.2. Modeling Results

**Figure 7:**
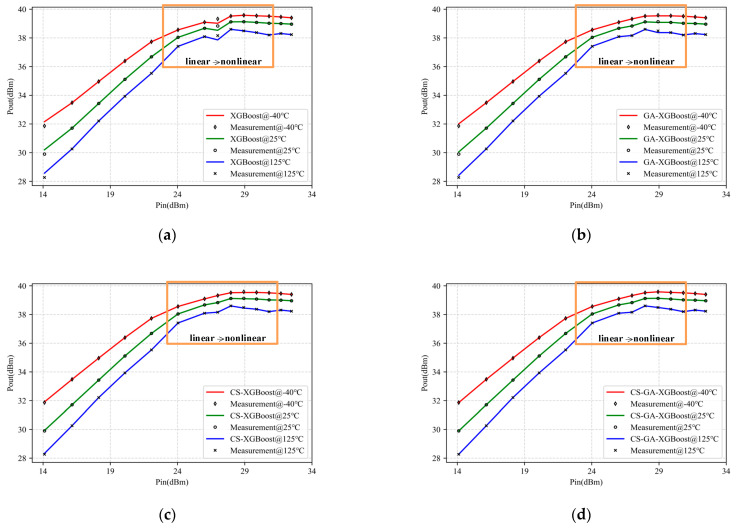
Modeling results of 2.5-GHz-GaN PA under −40 °C, 25 °C, 125 °C with four different models: (**a**) XGBoost; (**b**) GA-XGBoost; (**c**) CS-XGBoost; (**d**) CS-GA-XGBoost.

Figure 7 shows the modeling results using XGBoost, GA-XGBoost, CS-XGBoost, and CS-GA-XGBoost based on the measured input and output data of 2.5-GHz-GaN class-E PA under different temperatures. Table 1 provides the training MSE, validation MSE, and modeling time based on the above models. Among them, training MSE and validation MSE were reflections of model accuracy. The smaller the MSE is, the higher the modeling accuracy is. Modeling time reflects modeling speed. A shorter modeling time is a faster modeling speed.

The hyperparameters max_depth, learning_rate, and n_estimators greatly influence the modeling performance, so it was necessary to determine the optimal hyperparameter values before conducting modeling training. A traditional grid search method uses GA, CS, and CS-GA to search hyperparameters. The hyperparameter iterations for each algorithm were 30. In Table 1, we give the final optimal hyperparameters of each algorithm.

As shown in Figure 7a,b, when the data transitions from linear to nonlinear (as shown in the orange box), that is, when the output power approached saturation, XGBoost and GA-XGBoost exhibited significant fitting errors, indicating that the fitting accuracy of XGBoost and GA-XGBoost was poor. As shown in Figure 7c,d, CS-XGBoost and CS-GA-XGBoost exhibited good fitting performance. Based on the data in Table 1, the training and validation the MSE of CS-XGBoost and CS-GA-XGBoost were much better than those of XGBoost and GA-XGBoost. This is because CS has a strong global search ability, which enables it to search for the optimal parameters of the model more accurately. However, the global search capability of CS comes at the cost of longer modeling time, which is one order of magnitude higher than GA-XGBoost. This paper combined CS and GA, taking their respective advantages.

On the one hand, it can expand the size of the bird nest population and retain the strong global search ability of CS. On the other hand, it reduces the bird nest population’s search scope and shortens modeling time. Therefore, according to the data in the table, compared to XGBoost, GA-XGBoost, and CS-XGBoost, CS-GA-XGBoost had improved modeling accuracy by one order of magnitude or more while shortening modeling time by one order of magnitude or more.

To further demonstrate the superiority of the CS-GA-XGBoost proposed in this paper, classic machine learning algorithms, gradient boosting, random forest, and SVR, were also selected for comparison. Among them, the hyperparameters in gradient boosting, random forest, and SVR were all determined through a grid search. A grid search is a parameter searching method that uses exhaustive search. Among all candidate hyperparameter selections, by iterating and representing each possibility, the best-performing parameter to be found is the hyperparameter [32]. For gradient boosting, the hyperparameters found were the max_depth of decision trees, learning_rate, and the number of trees n_estimators. For random forest, the hyperparameters to be found were the number of decision trees n_estimators and the max_depth of the decision tree. For SVR, the hyperparameters found were the penalty parameter *c* and the kernel function coefficient *γ.* In the experiment, the hyperparameter iterations for each algorithm were 30. In Table 2, we give the final optimal hyperparameters of each algorithm.

**Figure 8:**
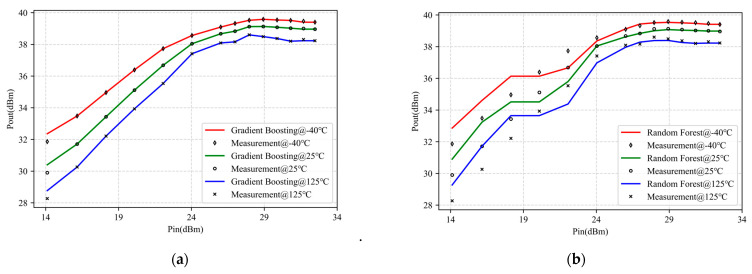

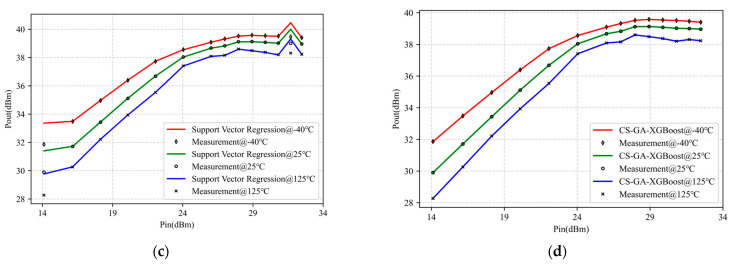
Modeling results of 2.5-GHz-GaN PA under −40 °C, 25 °C, 125 °C with four different models: (**a**) Gradient Boosting; (**b**) Random Forest; (**c**) Support Vector Regression (SVR); (**d**) CS-GA-XGBoost.

Figure 8 shows the modeling results based on the four algorithms mentioned above. Table 2 provides the corresponding training MSE, validation MSE, and modeling time.

As shown in Figure 8a–c, random forest exhibited significant segmentation characteristics during the modeling process, and its modeling accuracy was not as good as gradient boosting, which is consistent with the results in [14]. However, gradient boosting had the best modeling accuracy among the three, but it still showed deviations at some data turning points. Based on the data in Table 2, compared to gradient boosting, random forest, and SVR, CS-GA-XGBoost improved modeling accuracy by three orders of magnitude or more and shortened modeling time by two orders of magnitude, showing the excellent modeling results of CS-GA-XGBoost.

**Figure 9:**
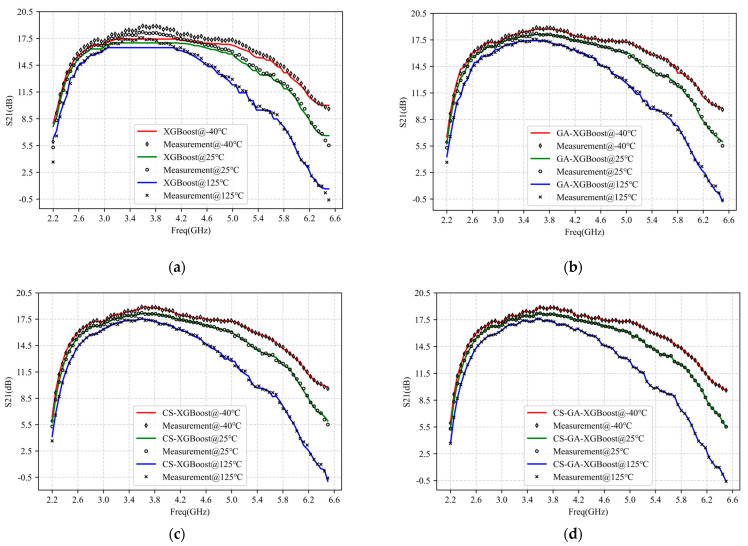
Modeling results of S_21_ of 2.2–6.5 GHz PA−40 °C, 25 °C, 125 °C with four different models: (**a**) XGBoost; (**b**) GA-XGBoost; (**c**) CS-XGBoost; (**d**) CS-GA-XGBoost.

S_21_ is the forward transmission coefficient commonly used to characterize the small signal gain of PA. Here, S_21_ was also selected for modeling to demonstrate the proposed CS-GA-XGBoost’s superiority further. Figure 9 and Table 3 show the results of modeling S_21_ of PA operating under −40 °C, 25 °C, and 125 °C with bandwidth ranging from 2.2 to 6.5 GHz by XGBoost, GA-XGBoost, CS-XGBoost, and CS-GA-XGBoost. The hyperparameter iterations for each algorithm were 20. To show clearly, we present 80 data points in the figures. The figures and tables show that XGBoost, which used traditional grid search techniques for hyperparameters optimization, exhibited the poorest modeling accuracy and speed. This is because a grid search limits the searching range of parameters, affecting modeling accuracy, and using enumeration will significantly increase modeling time [32]. GA-XGBoost performs well in modeling speed due to its fast convergence speed [27], but in terms of modeling accuracy, GA is prone to falling into local optima, affecting modeling accuracy [27]. CS-XGBoost exhibited high modeling accuracy as its convergence speed is not sensitive to parameter changes and is not easily trapped in local optima [29]. However, due to the slow convergence speed of CS, the modeling speed of CS-XGBoost was also slow [29]. Compared to XGBoost, GA-XGBoost, and CS-XGBoost, the proposed CS-GA-XGBoost improved the accuracy of the model by two orders of magnitude, or more, while also increasing the modeling speed by one order of magnitude or more. It is because CS-GA integrates GA’s crossover operators into CS, making full use of the global solid search ability of CS and the fast rate of convergence.

**Figure 10:**
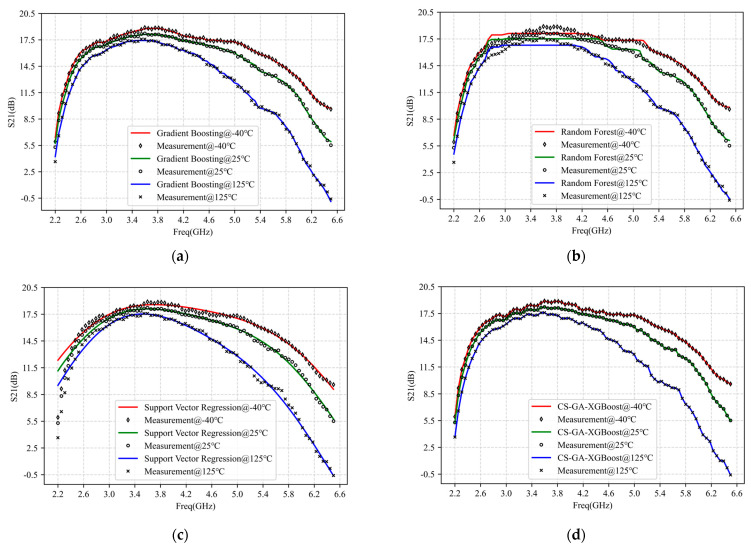
Modeling results of S_21_ at 2.2–6.5 GHz PA−40 °C, 25 °C, 125 °C with four different models: (**a**) Gradient Boosting; (**b**) Random Forest; (**c**) Support Vector Regression (SVR); (**d**) CS-GA-XGBoost.

Figure 10 and Table 4 show the results of modeling S_21_ of PA operating under −40 °C, 25 °C, and 125 °C with bandwidth ranging from 2.2 to 6.5 GHz by gradient boosting, random forest, SVR, and CS-GA-XGBoost. The hyperparameter iterations for each algorithm were 20. Among them, the optimal hyperparameters of gradient boosting, random forest, and SVR were still determined by grid search and presented in Table 4. The figure and table show that random forest and SVR exhibited poorer modeling accuracy and speed, especially when there was a turning point in the data point. It is easy to show significant fitting errors.

In contrast, the modeling accuracy and speed of gradient boosting have been improved, consistent with the conclusion in [14]. However, as shown in Figure 10a, gradient boosting cannot accurately fit every data point, and some data points may deviate slightly. Compared to gradient boosting, random forest, and SVR, the proposed CS-GA-XGBoost can improve modeling accuracy by three orders of magnitude or more while also increasing modeling speed by two orders of magnitude.

By fitting the Pin–Pout and S_21_ data under different temperatures, the superiority in modeling accuracy and speed of the proposed CS-GA-XGBoost was demonstrated.

## 4. Conclusions

This paper proposed a PA modeling method based on CS-GA-XGBoost. CS-GA integrates GA’s crossover operator into CS, making full use of the strong global search ability of CS and the fast rate of convergence of GA so that the improved CS-A can expand the size of bird nest populations while reducing the search range of bird nest population, and attain better optimization ability and rate of convergence. The experimental results showed that compared to XGBoost, GA-XGBoost, and CS-XGBoost, the proposed CS-GA-XGBoost improved the modeling accuracy by one order of magnitude or more and shorten the modeling time by one order of magnitude or more. In addition, compared to classic machine learning algorithms, including gradient boosting, random forest, and SVR, the proposed CS-GA-XGBoost improved modeling accuracy by three orders of magnitude or more and shortened modeling time by two orders of magnitude, demonstrating the superiority of the CS-GA-XGBoost in terms of modeling accuracy and speed. The CS-GA-XGBoost model presented in this paper can also be used for modeling other microwave/RF devices or circuits.

## Figures and Tables

**Table 1:** Training and validation MSE and modeling time of XGBoost, GA-XGBoost, CS-XGBoost, and CS-GA-XGBoost of P_in_-P_out_ for PA at 2.5 GHz.

Temperature (°C)	Model	Training MSE	Validation MSE	Modeling Time (s)
−40	XGBoost (* md = 1; * lr = 0.10; * ns = 60)	1.82 × 10^−2^	1.27 × 10^−2^	13.91
	GA-XGBoost (md = 2; lr = 0.20; ns = 59)	2.06 × 10^−3^	1.74 × 10^−3^	5.331
	CS-XGBoost (md = 3; lr = 0.25; ns = 61)	3.58 × 10^−4^	3.01 × 10^−4^	12.88
	CS-GA-XGBoost (md = 3; lr = 0.28; ns = 57)	3.75 × 10^−5^	3.14 × 10^−5^	0.309
25	XGBoost (md = 1; lr = 0.31; ns = 32)	1.73 × 10^−2^	1.39 × 10^−2^	12.89
	GA-XGBoost (md = 2; lr = 0.20; ns = 60)	1.85 × 10^−3^	1.78 × 10^−3^	6.339
	CS-XGBoost (md = 3; lr = 0.27; ns = 50)	2.01 × 10^−4^	2.13 × 10^−4^	11.17
	CS-GA-XGBoost (md = 3; lr = 0.33; ns = 45)	4.23 × 10^−5^	4.68 × 10^−5^	0.299
125	XGBoost (md = 1; lr = 0.15; ns = 45)	1.55 × 10^−2^	1.47 × 10^−2^	16.39
	GA-XGBoost (md = 2; lr = 0.19; ns = 58)	1.73 × 10^−3^	2.70 × 10^−3^	5.279
	CS-XGBoost (md = 3; lr = 0.20; ns = 25)	3.73 × 10^−4^	3.08 × 10^−4^	12.01
	CS-GA-XGBoost (md = 3; lr = 0.3; ns = 33)	5.06 × 10^−5^	5.61 × 10^−5^	0.339

* (md: max_depth; or: learning_rate; ns: n_estimators).

**Table 2:** Training and validation of the MSE and modeling time of gradient boosting, random forest, support vector regression, and CS-GA-XGBoost of P_in_-P_out_ for PA in 2.5 GHz.

Temperature (°C)	Model	Training MSE	Validation MSE	Modeling Time (s)
−40	Gradient Boosting (md = 4; or = 82; ns = 0.15)	1.14 × 10^−2^	1.81 × 10^−2^	10.46
	Random Forest (md = 3; ns = 55)	1.98 × 10^−1^	3.51 × 10^−1^	10.76
	SVR (c = 32; *γ* = 0.11)	2.99 × 10^−1^	2.32 × 10^−1^	11.99
	CS-GA-XGBoost (* md = 3; * lr = 0.28; * ns = 57)	3.75 × 10^−5^	3.14 × 10^−5^	0.309
25	Gradient Boosting (md = 3; lr = 115; ns = 0.11)	1.10 × 10^−2^	1.34 × 10^−2^	10.83
	Random Forest (md = 2; ns = 81)	3.84 × 10^−1^	4.04 × 10^−1^	13.21
	SVR (c = 45; *γ* = 0.10)	2.01 × 10^−1^	2.33 × 10^−1^	13.90
	CS-GA-XGBoost (md = 3; lr = 0.33; ns = 45)	4.23 × 10^−5^	4.68 × 10^−5^	0.299
125	Gradient Boosting (md = 3; lr = 120; ns = 0.18)	1.71 × 10^−2^	1.97 × 10^−2^	10.36
	Random Forest (md = 3; ns = 62)	3.46 × 10^−1^	4.92 × 10^−1^	10.97
	SVR (c = 49; *γ* = 0.15)	2.13 × 10^−1^	2.04 × 10^−1^	14.43
	CS-GA-XGBoost (md = 3; lr = 0.3; ns = 33)	5.06 × 10^−5^	5.61 × 10^−5^	0.339

* (md: max_depth; lr: learning_rate; ns: n_estimators).

**Table 3:** Training and validation MSE and modeling time of XGBoost, GA-XGBoost, CS- XGBoost, and CS-GA-XGBoost of S_21_ of PA with 2.2–6.5 GHz.

Temperature (°C)	Model	Training MSE	Validation MSE	Modeling Time (s)
−40	XGBoost (* md = 2; * lr = 0.10; * ns = 32)	4.42 × 10−1	4.82 × 10−1	191.3
	GA-XGBoost (md = 3; lr = 0.19; ns = 32)	1.42 × 10−2	2.82 × 10−2	34.87
	CS-XGBoost (md = 5; lr = 0.28; ns = 71)	9.51 × 10−3	9.76 × 10−3	188.5
	CS-GA-XGBoost (md = 5; lr = 0.28; ns = 71)	3.08 × 10−5	1.65 × 10−5	6.582
25	XGBoost (md = 2; lr = 0.12; ns = 28)	3.03 × 10−1	3.74 × 10−1	188.3
	GA-XGBoost (md = 3; lr = 0.23; ns = 38)	1.27 × 10−2	2.64 × 10−2	36.87
	CS-XGBoost (md = 5; lr = 0.35; ns = 58)	9.56 × 10−3	9.88 × 10−3	169.8
	CS-GA-XGBoost (md = 5; lr = 0.35; ns = 58)	2.69 × 10−5	1.72 × 10−5	5.485
125	XGBoost (md = 2; lr = 0.15; ns = 25)	2.18 × 10−1	2.96 × 10−1	189.2
	GA-XGBoost (md = 3; lr = 0.29; ns = 31)	3.14 × 10−2	4.19 × 10−2	33.87
	CS-XGBoost (md = 4; lr = 0.38; ns = 35)	9.53 × 10−3	0.86 × 10−3	170.8
	CS-GA-XGBoost (md = 5; lr = 0.31; ns = 65)	3.87 × 10−5	3.02 × 10−5	6.183

* (md: max_depth; lr: learning_rate; ns: n_estimators).

**Table 4:** Training and validation MSE and modeling time of gradient boosting, random forest, support vector regression, and CS-GA-XGBoost of S_21_ of PA with 2.2–6.5 GHz.

Temperature (°C)	Model	Training MSE	Validation MSE	Modeling Time (s)
−40	Gradient Boosting (* md = 3; * lr = 0.17; * ns = 71)	1.94 × 10−2	1.41 × 10−2	129.3
	Random Forest (md = 5; ns = 31)	1.16 × 10−1	1.21 × 10−1	189.4
	SVR (c = 22; *γ* = 0.11)	5.17 × 10−1	5.64 × 10−1	196.3
	CS-GA-XGBoost (md = 5; lr = 0.28; ns = 71)	3.08 × 10−5	1.65 × 10−5	6.582
25	Gradient Boosting (md = 3; lr = 0.15; ns = 82)	1.84 × 10−2	176 × 10−2	126.9
	Random Forest (md = 5; ns = 18)	1.17 × 10−1	1.33 × 10−1	197.2
	SVR (c = 18; *γ* = 0.16)	5.44 × 10−1	6.83 × 10−1	194.3
	CS-GA-XGBoost (md = 5; lr = 0.35; ns = 58)	2.69 × 10−5	1.72 × 10−5	5.485
125	Gradient Boosting (md = 3; lr = 0.12; ns = 58)	1.54 × 10−2	1.32 × 10−2	125.9
	Random Forest (md = 5; ns = 22)	1.27 × 10−1	1.37 × 10−1	189.2
	SVR (c = 11; *γ* = 0.21)	5.06 × 10−1	7.50 × 10−1	195.3
	CS-GA-XGBoost (md = 5; lr = 0.31; ns = 65)	3.87 × 10−5	3.02 × 10−5	6.183

* (md: max_depth; lr: learning_rate; ns: n_estimators).

## Data Availability

Not applicable.
